# Barriers and Facilitation Measures Related to People With Mental Disorders When Using the Web: A Systematic Review

**DOI:** 10.2196/jmir.5442

**Published:** 2016-06-09

**Authors:** Renaldo Bernard, Carla Sabariego, Alarcos Cieza

**Affiliations:** ^1^ Department of Medical Informatics, Biometry and Epidemiology – IBE Chair for Public Health and Health Services Research, Research Unit for Biopsychosocial Health Ludwig-Maximilians-Universität München Munich Germany; ^2^ Blindness and Deafness Prevention, Disability and Rehabilitation (BDD) World Health Organization Geneva Switzerland

**Keywords:** World Wide Web, mental disorders, systematic review, accessibility, interaction design, Web-based interaction

## Abstract

**Background:**

Mental disorders (MDs) affect almost 1 in 4 adults at some point during their lifetime, and coupled with substance use disorders are the fifth leading cause of disability adjusted life years worldwide. People with these disorders often use the Web as an informational resource, platform for convenient self-directed treatment, and a means for many other kinds of support. However, some features of the Web can potentially erect barriers for this group that limit their access to these benefits, and there is a lack of research looking into this eventuality. Therefore, it is important to identify gaps in knowledge about “what” barriers exist and “how” they could be addressed so that this knowledge can inform Web professionals who aim to ensure the Web is inclusive to this population.

**Objective:**

The objective of this study was to provide an overview of existing evidence regarding the barriers people with mental disorders experience when using the Web and the facilitation measures used to address such barriers.

**Methods:**

This study involved a systematic review of studies that have considered the difficulties people with mental disorders experience when using digital technologies. Digital technologies were included because knowledge about any barriers here would likely be also applicable to the Web. A synthesis was performed by categorizing data according to the 4 foundational principles of Web accessibility as proposed by the World Wide Web Consortium, which forms the necessary basis for anyone to gain adequate access to the Web. Facilitation measures recommended by studies were later summarized into a set of minimal recommendations.

**Results:**

A total of 16 publications were included in this review, comprising 13 studies and 3 international guidelines. Findings suggest that people with mental disorders experience barriers that limit how they perceive, understand, and operate websites. Identified facilitation measures target these barriers in addition to ensuring that Web content can be reliably interpreted by a wide range of user applications.

**Conclusions:**

People with mental disorders encounter barriers on the Web, and attempts have been made to remove or reduce these barriers. As forewarned by experts in the area, only a few studies investigating this issue were found. More rigorous research is needed to be exhaustive and to have a larger impact on improving the Web for people with mental disorders.

## Introduction

Mental disorders (MDs) are a significant public health issue owing to their high impact on people with these disorders, in terms of restrictions placed on their participation in all areas of life, family life and the wider society. Mental disorders affect almost 1 in 4 adults at some point during their lifetime [1] and coupled with substance use disorders are the fifth leading cause of disability adjusted life years worldwide [2]. People with mental disorders (PwMD) often experience similar impairments, activity limitations, and restricted participation in life events, even with the diversity in symptoms and etiology associated with these conditions [3]. Family members often provide care, which sometimes puts a strain on familial relationships, reduces opportunities for leisure, and negatively impacts finances due to time spent providing care instead of working [4]. The associated reduction in productivity from both affected persons and their family can translate to a decrease in contributions to the local economy [5]. In addition, having a large segment of the population subscribing to treatment and support services incurs considerable costs [5].

The Web is often used as a source of support for PwMD and shows great promise for the reduction of the burden of mental disorders. Mental health–related Web browsing, primarily for information seeking, is common among PwMD [6,7]. Web-based mental health communities are known to supplement traditional mental health services [8] and act as an important factor in encouraging PwMD to seek professional help [9]. A recent meta-analysis has indicated that guided Web-based cognitive behavioral therapy may be as effective as the face-to-face equivalent for social anxiety disorder, panic disorder, spider phobia, and depressive symptoms [10]. Many other Web-based treatment and intervention options are increasingly being explored for other mental disorders (eg, posttraumatic stress disorder, eating disorders) [11] and populations including children (eg, Project CATCH-IT, MoodGYM) [12,13] with positive results.

There are also features of the Web environment that could potentially limit how much PwMD who experience cognitive deficits can benefit from the Web. Using the Web is considered a very cognitively demanding activity requiring not only good knowledge and understanding of Web features (eg, search engines) but also the ability to quickly analyze, synthesize, evaluate, and apply presented information while avoiding inconsequential details (eg, adverts and untrustworthy information) that are abundant on the Web [14]. Several cognitive domains, including executive functioning, attention, and memory, are commonly impaired in PwMD [15]. These impairments may be linked to difficulties using the Web such as when performing Web searches, task switching, retaining and recalling information, and ignoring distractions (eg, adverts) to focus attention. Moreover, the Web has also been found to be relatively absent of nonverbal and social context cues (eg, gestures, facial expression) compared with off-line [16,17]. These cues are important for guiding behavior when interacting with others, and their absence could make social interaction difficult. Although Web users are normally able to skillfully compensate and overcome these “deficiencies” [18], sometimes even by capitalizing on them [19], it could be challenging for PwMD who experience cognitive deficits to do the same.

People with mental disorders have received little attention from Web accessibility research despite increased inquiries into the difficulty others with cognitive impairment face on the Web. This research gap was highlighted over a decade ago [20,21], and more recently, there has been some indication that the gap still exists [22]. Current recommendations also prescribe the same treatment to address accessibility for PwMD and a myriad of other diverse conditions that fall under the broad heading of conditions associated with cognitive limitations (eg, intellectual disabilities, multiple sclerosis) [23].

A comprehensive review of literature concerned with the barriers PwMD encounter when using the Web and/or the facilitation measures developed to address these barriers is needed to ensure that the Web is inclusive to this population. Available knowledge will support Web professionals in making well-informed choices about the removal of barriers affecting PwMD. If this is not possible, it may instead provide facilitation measures to accommodate this group. As a result, Web-based resources could be systematically evaluated for compliance with measures that are known to remove barriers or provide facilitation for PwMD. Identified gaps in knowledge about “what” barriers exist and “how” they could be addressed—based on a comparison and integration of what is known on the topic—is likely to encourage further research into these highlighted areas as well.

The objective of this systematic review was to provide an overview of the existing evidence regarding the barriers PwMD experience when using the Web and facilitation measures used to address such barriers. Specific aims are to detail barriers and facilitation measures, how they were identified or developed, and related trends (ie, the extent of coverage for specific mental disorders or digital technologies, study designs used, publication recency, and research region).

## Methods

A systematic review was carried out to identify barriers PwMD encounter when using the Web and the recommended facilitation measures to remove or reduce these barriers.

### Search Strategy

Search terms were broadly based on concepts relating to Web accessibility, mental disorders, and also digital technologies (see Multimedia Appendix 1). Digital technologies were included because knowledge about any barriers here would likely be also applicable to the Web. This was also a proactive measure to avoid having the review suffer from the paucity of research in the area as revealed by preliminary searches. Databases searched include MEDLINE, PsycARTICLES, CINAHL, Library, Information Science &Technology Abstracts, Computers & Applied Sciences Complete, Inspec, Web of Science Core Collection. Reference lists of included publications were also searched to avoid missing relevant publications not identified during the search of databases. There were no publication date restrictions to ensure that the review included as many studies as possible. There was also no restriction to empirical studies. Other types of publications such as international standards and guidelines are usually widely adopted and highly regarded and can be especially helpful when there is insufficient empirical evidence on a particular issue.

### Eligibility Criteria

Included publications describe the difficulties PwMD encounter when using any digital technology or provide guidance on how to improve the accessibility of any digital technology for this group. All mental disorders were considered regardless of a formal diagnosis or not. All digital technologies such as computers, video games, mobile devices, and websites were also considered. Journal articles, gray literature, international and national standards and guidelines, reports, and conference proceedings written in the English language were considered for inclusion. Publications in the form of commentaries, letters to the editors, and editorials were excluded.

### Eligibility Assessment

One reviewer (RB) screened all abstracts, and another (DH) screened 84% (1692/2013) selected at random. Both screenings were conducted independently to reduce the chance of reviewer bias and increase reliability [24]. Inconsistences in ratings—eligible, ambiguous, or excluded—were later discussed and resolved by consensus. One reviewer (RB) then appraised the full texts of abstracts rated as eligible.

### Data Extraction and Synthesis of Results

Information extracted from studies was study characteristics—publication year, country, study design, methods and participants or target population (eg, mental disorders, age, gender, and education); barriers and facilitation measures—process used for the development of the facilitation measure and related mental disorders; and definitions of accessibility and disability. Data extracted from other documents—international standards and guidelines—did not include information about study designs and participants (eg, age and gender).

The International Classification of Functioning, Disability and Health was used to define barriers and facilitation measures [25]. Factors (eg, small font, complicated language) that through their absence or presence limit functioning were identified as barriers. Conversely, factors (eg, legible font, simple language) that instead improve functioning through their absence or presence were identified as facilitation measures.

Synthesis was performed by categorizing all findings and later summarizing facilitation measures recommended by studies. Data were first categorized according to the 4 foundational principles of Web accessibility: operable—user interface components and navigation must be easy and safe to use; understandable—information and the operation of a user interface must be easily interpreted accurately; perceivable—information and user interface components must be presentable to users in ways they can be sufficiently aware on these components; robust—content must be flexible enough that a wide range of user agents, including technologies that enable persons with disabilities to perform tasks that would be otherwise challenging (ie, assistive technologies), can interpret it reliably [26]. These 4 foundational principles were proposed by the World Wide Web Consortium (W3C) and form the necessary basis for anyone to gain adequate access to the Web. Results from studies came from 2 sources—expert opinion or empirical research—and they are labeled to denote these different sources. Facilitation measures from guidelines are also labeled for easy identification. Facilitation measures recommended by studies were later summarized into a set of minimal recommendations after the categorization of findings. Those from guidelines have already been aptly summarized elsewhere [27-29].

## Results

**Figure 1:**
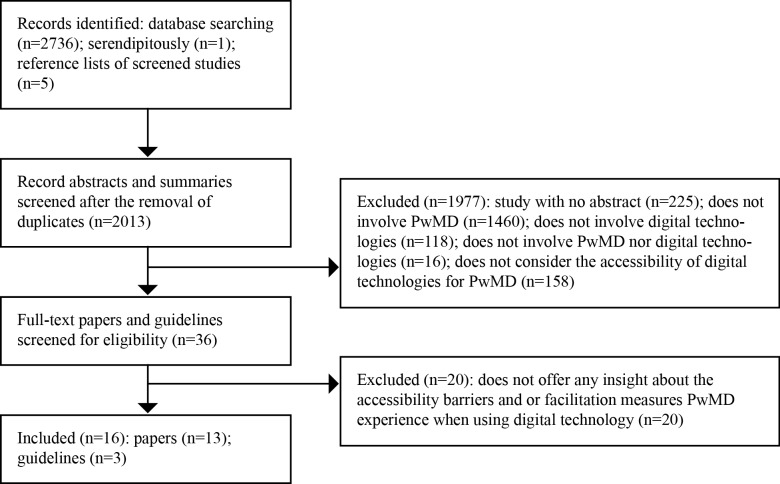
Flowchart of the review identification and selection process.

A total of 16 publications were included in this review, comprising 13 studies reporting on the usability of various technologies [30-40] and Internet or computer use among PwMD [41,42] and 3 international guidelines [23,43,44], which were all developed by the W3C. These guidelines have been adopted by many governments and are also widely considered as the international standard for Web accessibility. A flow chart of the review process is presented in Figure 1.

### Study and Guideline Characteristics

Nine of the included studies [30-33,35,37-39,42] originated in the United States, 2 studies [34,40] in the United Kingdom, one [41] in Austria, and another [36] in Sweden as summarized in Table 1. Over 62% (10/16) of the included publications [32-34,37,38,42] were published within the last 5 years, and the earliest [35] was published in 1998.

All 3 included guidelines were published by the W3C based in the United States. However, the guidelines are the result of collaboration among international experts. Two of the three included guidelines (User Agent Accessibility Guidelines 1.0 and Authoring Tool Accessibility Guidelines 1.0) were published over 12 years ago, and the third (Web Content Accessibility Guidelines 2.0) was published in 2008.

**Table 1:** Characteristics of included publications.

Citation, sample size (n), and year	Origin country	Study design	Digital technology	Diagnosis
[35], 52, 1998	United States	Qualitative, focus groups and interviews, clustering and summation	Multimedia application	Depression
[43], 2000	United States	Guideline	Web	Mental disorders
[31], 5, 2002	United States	Qualitative, focus group and usability testing, content analysis	Website	Anxiety disorder and depression
[44], 2002	United States	Guideline	Web	Mental disorders
[38], 98, 2007	United States	Quantitative, usability testing	Website	Bipolar disorder, schizophrenia, schizoaffective disorder, depression
[23], 2008	United States	Guideline	Web	Mental disorders
[41], 26, 2010	Austria	Qualitative, interviews, content analysis	Internet and website	Schizophrenia, schizoaffective disorder^a^
[32] (n=16), 2011	United States	Mixed, interviews, usability testing and expert review, thematic analysis, and descriptive statistics	Website	Schizophrenia, bipolar disorder, depression
[33], 71, 2011	United States	Qualitative, interviews and usability Testing, descriptive statistics, and *t*-tests	Website	Severe mental illness
[39], 149, 2012	United States	Quantitative (fractional factorial experimental design), usability testing, polychotomous logistic regression, and mixed-effect regression	Website	Substance use disorder, schizophrenia, depression, bipolar disorder, other psychotic disorder, schizoaffective disorder, anxiety disorder^b^
[42], 28, 2013	United States	Qualitative, interviews and observations, thematic and task analysis	Computer and website	Schizophrenia, bipolar disorder, depression, anxiety disorder, schizoaffective disorder
[37], 38, 2013	United States	Quantitative, usability testing, linear mixed-effect regression	Website	Schizophrenia, schizoaffective disorder^b^
[40], 12, 2013	United Kingdom	Qualitative, focus group, thematic analysis	Website	Bipolar disorder^b^
[30], 924, 2013	United States Mixed, usability testing and survey, thematic analysis, descriptive statistics	Mobile phone and website	Schizophrenia, schizoaffective disorder	
[34], 20, 2014	United Kingdom	Qualitative, focus group, thematic analysis	Website	Depression, anxiety disorder
[36], ≥100, 2015	Sweden	Qualitative, focus group, thematic analysis Digital technologies	Bipolar disorder, depression, schizophrenia, anxiety disorder, mental disorders	

^a^ Diagnosis was established using the International Classification of Diseases, 10th revision.

^b^ Diagnosis was established using the Diagnostic and Statistical Manual of Mental Disorders, 4th edition.

### Design and Methods

Nine of the included studies investigated the usability of Web-based resources [30-34,37-40] and multimedia tools [35]. One study focused on Internet use [41], one on the use of digital technologies [36], one on the development of a mobile phone system [30], and another on computer use [42] among PwMD. Eight of the included studies used qualitative methods [31,33-36,40-42], 3 [37-39] adopted a quantitative approach, and 2 [30,32] used mixed methods. Seven studies used usability testing [30-33,37-39], 5 used interviews [32,33,35,41,42], 5 used focus groups [32,33,35,41,42], and single studies used observations [42], survey [30], and user testing.

The 3 included guidelines [23,43,44] were primarily developed based on contributions over several years from experts involved in international working groups on varying aspects of Web accessibility [45].

### Sample Characteristics

Sample sizes for included studies ranged from 5 to >100 (mean 48). Overall, 11 studies [30-33,36-42] reported the age of participants, which ranged from 18 to at least 75 years. Three studies [37,39,40] used the Diagnostic and Statistical Manual of Mental Disorders, 4th edition (DSM IV), 1 [41] used the International Classification of Diseases, 10th revision (ICD-10), and the remaining studies did not mention the use of a classification of mental disorders. Samples including people with schizophrenia (69%) [30,32,35-39,41,42] were most common among the 13 included studies, followed by samples where participants were affected by depression (62%) [31,32,34-36,38,39,42], schizoaffective disorder (46%) [30,37-39,41,42], anxiety disorders (38%) [31,34,36,39,42], and bipolar disorder (38%) [32,36,38,40,42]. Single studies reported that participants had severe mental illness (SMI) (eg, schizophrenia, schizoaffective disorder, bipolar disorder, and major depression) [33], mental disorders [36], psychotic disorders [39], and substance use disorder [39] but did not state any particular mental disorder. Most studies considered more than 1 mental disorder except [33], which focused on schizophrenia and [40] on bipolar disorder.

All 3 included guidelines were developed to give guidance on how to remove and reduce barriers experienced by people with a range of disabilities including auditory, cognitive, and neurological, physical, speech, and visual disabilities. Extracted guidelines were identified by the authors of the guidelines as being relevant to cognitive and neurological disorders [46]. These disorders include attention-deficit hyperactivity disorder, autism spectrum disorder, intellectual disabilities, learning disabilities, memory impairments, multiple sclerosis, perceptual disabilities, seizure disorders, and mental disorders. No particular mental disorder was specified.

### Digital Technology

As summarized in Table 1, websites were the most studied digital technology, followed by single studies each investigating either computers [42] or multimedia applications [35]. Only three studies [42,30,36] investigated more than 1 technology, viz computers and websites, mobile phone and websites, and several digital technologies, respectively. The 3 included guidelines target websites (ie, Web Content Accessibility Guidelines 2.0), user agents (ie, any software that retrieves, renders, and facilitates end user interaction with Web content; User Agent Accessibility Guidelines 1.0) and Web authoring tools (Authoring Tool Accessibility Guidelines 1.0).

### Scope of Barriers and Facilitation Measures Related to Digital Technology Usage by PwMD

Included studies revealed 42 barriers and 59 facilitation measures. These are summarized in Tables 2 and 5. Four studies [31,32,35,37] did not mention any barriers and 2 [36,41] no facilitation measures. Four studies [30,33,34,38] recommended facilitation measures to address barriers, and only 25 of these pairings were identified.

The 3 included guidelines recommended 30 facilitation measures and did not explicitly report any barriers. However, the W3C has published several barriers on its website that people with cognitive and neurological disabilities including mental health disabilities face when using the Web. Examples of these barriers include complex navigation mechanisms, page layouts that are difficult to understand and use, and moving, blinking, or flickering content, and background audio that cannot be turned off [46].

Of the 131 identified barriers and facilitation measures, 63 were relevant to depression (48%), 54 to schizophrenia (41%), 48 to anxiety disorders (37%), 39 to bipolar disorder (30%), 37 to mental disorders (28%), 35 to schizoaffective disorder (27%), 11 to SMI (8%), and 3 to substance abuse and psychotic disorders equally (2%). Most of the 42 identified barriers were relevant to people with depression (64%), followed by those with an anxiety disorder (62%), schizophrenia (50%), bipolar disorder (40%), schizoaffective disorder (31%), mental disorders (17%), SMI (12%), and substance use disorder and other psychotic disorders equally (2%). Identified facilitation measures (n=89) mostly targeted people with depression (40%), schizophrenia (37%), mental disorders (34%), and anxiety, bipolar disorder and schizoaffective disorder equally (25%). SMI (7%) and substance use disorder and other psychotic disorders equally (2%) accounted for a small portion of the identified facilitation measures.

All barriers identified were revealed by research findings. Identified facilitation measures were proposed directly from research findings (n=31) [30,33,37-40], by international working groups of experts in the area of accessibility (n=30) [23,43,44] and expert opinion of researchers conducting studies (n=28) [31,32,34,35,42].

**Table 2:** Barriers and facilitation measures categorized by the ‘perceivable’ foundational principle of Web accessibility.

Barrier	Facilitation measure
Unable to locate information [34]	Provide intuitive navigation and ensure information filters and search functions work properly^a^.
Nonperceivable icons [34]	Avoid complicated language and ensure menu options and links are easy to understand^a^.
Too small font [30]	Increase font size^b^.
Difficulty reading small font and with eye strain [42]	Use small but legible font and refrain from using graphics in websites with shallow information hierarchies that do not feature navigational lists^b^ [39]. Use large navigation buttons^a^ [32]. Use a minimal number of colors that differentiates information and contrasts well^a^ [31]. Use a simple design with pages that are pleasing to the eye and easy to read^a^ [31]. Use graphics that are purposeful to the website^a^ [31]. Prominently present hyperlinks: ensure clear labeling and highly visible positioning^b^ [37]. Make hyperlinks' text as explicit as possible^b^ [37]. List hyperlinks for a given topic together in a single column^b^ [37]. Font size, buttons, and links should be sufficiently large to ensure usability^a^ [42]. Use attention grabbing and not boring design^b^ [40]. Guideline 1.1: Provide text alternatives for any nontext content so that it can be changed into other forms people need, such as large print, braille, speech, symbols, or simpler language^c^ [23]. Guideline 1.2: Provide alternatives for time-based media^c^ [23]. Guideline 1.3: Create content that can be presented in different ways (eg, simpler layout) without losing information or structure^c^ [23]. Guideline 1.4: Make it easier for users to see and hear content including separating foreground from background^c^ [23]. Guideline 5: Ensure that the user can control the behavior of viewports (ie, screen) and user interface controls, including those that may be manipulated by the author (eg, through scripts—list of computer commands)^c^ [44]. Guideline 3: Support the creation of accessible content^c^ [43]. Guideline 2: Generate standard markup (ie, document annotations)^c^ [43]. Guideline 1: Support accessible authoring practices^c^ [43]. Guideline 7: Ensure that the authoring tool is accessible to authors with disabilities^c^ [43]. Guideline 2: Ensure that users have access to all content, notably conditional content that may have been provided to meet the requirements of the Web Content Accessibility Guidelines 1.0^c^ [44]. Guideline 3: Ensure that the user may turn off rendering of content (eg, audio, video, scripts) that may reduce accessibility by obscuring other content or disorienting the user^c^ [44]. Guideline 4: Ensure that the user can select preferred styles (eg, colors, size of rendered text, and synthesized speech characteristics) from choices offered by the user agent. Allow the user to override author-specified and user agent default styles^c^ [44]. Guideline 11: Allow users to configure the user agent so that frequently performed tasks are made convenient and allow users to save their preferences^c^ [44].

^a^Facilitation measure derived from expert opinion of researcher(s) conducting a study.

^b^Facilitation measure derived from empirical evidence.

^c^Facilitation measure derived from working group of experts.

**Table 3:** Barriers and facilitation measures categorized by the ‘understandable’ foundational principle of Web accessibility.

Barrier	Facilitation measure
Information overload [34]	Ensure information is organized well and avoids distracting design^a^.
Poor organization and presentation [34]	Ensure information is organized well and avoids distracting design^a^.
Excessive advertisements [34]	Ensure information is organized well and avoids distracting design^a^.
Confusing menu options [34]	Avoid complicated language and ensure menu options and links are easy to understand^a^.
Complicated language [34]	Avoid complicated language and ensure menu options and links are easy to understand^a^.
Complex purchasing process [34]	Avoid complicated language and ensure menu options and links are easy to understand^a^.
Distracting design [34]	Ensure information is organized well and avoids distracting design^a^.
Use of abstract reasoning [38]	Present text at a low reading level^b^.
Difficulty comprehending text [33]	Present text in large font and language below a fifth-grade reading level^b^.
Difficulty understanding abbreviations [30]	Remove abbreviations^b^.
Difficulty understanding long words [30]	Reduce text^b^.
Too lengthy text [30]	Simplify wording to fourth-grade level^b^.
Overabundance of information [41]	
Unwanted movements or flickering [36]	
Cluttered design [36]	
Lack of logic and consequence in concept and design [36]	Provide resources in video and audio format^a^ [35]. Use a modular and hierarchical approach when presenting information^a^ [35]. Present important information first^a^ [35]. Use large navigation buttons^a^ [32]. Provide explicit labels that use longer concrete phrases to describe content^a^ [32]. Explicit instructions on how to use the website^a^ [32]. Provide text at fifth-grade reading level^a^ [32]. Provide instructions on how to navigate programs and websites^a^ [42]. Use a simple design with pages that are pleasing to the eye and easy to read^a^ [31]. Provide category headings that clearly identify what information is underneath^a^ [31]. Use menus with options that are ordered in a meaningful way and/or have an evident hierarchy^a^ [31]. Give a clear identity to the homepage^a^ [31]. Provide a homepage with just the right amount of information (graphics, text, links) to make the page understandable without overwhelming the user^a^ [31]. Use language that the user can identify with^a^ [31]. Meaningfully group of information^a^ [31]. Use graphics that are purposeful to the website^a^ [31]. Comprehensively list hyperlinks surrounding a given topic^b^ [37]. Include minimal amount of content on pages^b^ [37]. Single topic of interest: group hyperlinks and topics in one area of the screen^b^ [37]. List hyperlinks for a given topic together in a single column^b^ [37]. Use an ample number of images and visual aids^b^ [30]. Provide content users can identify with (eg, case stories, worked examples, and success stories)^b^ [40].
	Use a flat hierarchy^b^ [38]. Provide explicit labeling^b^ [38]. Use lower-level modules (eg, code and data to implement a specific functionality)^b^ [38]. Use familiar phrasing^b^ [38]. Guideline 3.1: Make text content readable and understandable^c^ [23]. Guideline 3.2: Make Web pages appear and operate in predictable ways^c^ [23]. Guideline 3.3: Help users avoid and correct mistakes^c^ [23]. Guideline 7: Observe operating environment conventions for the user agent user interface, documentation, input configurations, and installation^c^ [44]. Guideline 12: Ensure that the user can learn about software features that benefit accessibility from the documentation. Ensure that the documentation is accessible^c^ [44]. Guideline 2: Ensure that users have access to all content, notably conditional content that may have been provided to meet the requirements of the Web Content Accessibility Guidelines 1.0^c^ [44]. Guideline 3: Ensure that the user may turn off rendering of content (eg, audio, video, scripts) that may reduce accessibility by obscuring other content or disorienting the user^c^ [44]. Guideline 4: Ensure that the user can select preferred styles (eg, colors, the size of rendered text, and synthesized speech characteristics) from choices offered by the user agent. Allow the user to override author-specified and user agent default styles^c^ [44]. Guideline 5: Integrate accessibility solutions into the overall “look and feel”^c^ [43]. Guideline 6: Promote accessibility in help and documentation^c^ [43]. Guideline 4: Provide ways of checking and correcting inaccessible content^c^ [43]. Guideline 1: Support accessible authoring practices^c^ [43]. Guideline 7: Ensure that the authoring tool is accessible to authors with disabilities^c^ [43]. Guideline 3: Support the creation of accessible content^c^ [43]. Guideline 2: Generate standard markup^c^ [43].

^a^Facilitation measure derived from expert opinion of researcher(s) conducting a study.

^b^Facilitation measure derived from empirical evidence.

**Table 4:** Barriers and facilitation measures categorized by the ‘operable’ foundational principle of Web accessibility.

Barrier	Facilitation measure
Poor navigation [34]	Provide intuitive navigation^a^.
Poor information filters [34]	Ensure filters and search functions work properly^a^.
Information overload [34]	Ensure information is organized well and avoid distracting design^a^.
Difficulty with fine motor coordination [33]	Change double clicking to single clicking^c^.
Difficulty clicking small radio buttons [33]	Change small buttons to large buttons^b^.
Difficulty using a mouse [33]	Create video mouse tutorial^b^.
Lack of knowledge on how to navigate a website [33]	Create basic instructions on how to change screens^b^.
Lack of knowledge on how to navigate a website [33]	Create a flat website (without multiple layers)^b^.
Too close and sensitive touchscreen buttons [30]	Enlarge buttons and space between them and require long enough touch-and-release functionality^b^.
Navigating a website with more than 5 hierarchical levels [39]	Use 99 words or less, 2 navigational areas or less, 7 hyperlinks or less, and few topic areas covered per page and no graphics and toolbars^b^.
Time-limited response forms [34]	
Slow response in websites loading information [34]	
Necessity to distance oneself from illness-related topics as part of the recovery process [41]	
Difficulty operating a computer mouse [42]	
Difficulty typing words in designated areas [42]	
Difficulty scrolling or using menu options to access information [42]	
Difficulty navigating [42]	
Processing delays [40]	
Broken links [40]	
Additional software requirements [40]	
Unwanted movements or flickering [36]	
Cluttered design [36]	
Evil design (when design is used to persuade or trick you to do something) [36]	
Functions and services with login [36]	
Lack of logic and consequence in concept and design [36]	
Lack of trustworthiness [36]	
Managing passwords and other codes (eg, Completely Automated Public Turing test to tell Computers and Humans Apart—CAPTCHA) [36]	Use a website with no more than 3 hierarchal levels and words per hyperlink and that has navigational lists^b^ [39]. Use small but legible font and refrain from using graphics in websites with shallow hierarchies that do not feature navigational lists^b^ [39]. Use of different media and technological additions (eg, reward logo or bookmark functionality)^b^ [40]. Ensure resource can be easily used by people with low computer literacy^b^ [40]. Allow users to progress through the system at their own pace^a^ [35]. Pop-up menus that appear with hovering to reduce need for clicking^a^ [32]. Use a shallow hierarchy (reach the destination within 2 clicks)^a^ [32].
	Use large navigation buttons^a^ [32]. Provide several options (eg, mouse, keyboard arrows, touch screen) to assist users when navigating programs and websites^a^ [42]. Provide instructions on how to navigate programs and websites^a^ [42]. Use shorter pages that do not require a lot of scrolling, especially for the home page^a^ [31]. Allow for personalization or getting the best fit^b^ [40]. Guideline 2.2: Provide users enough time to read and use the content^c^ [23]. Guideline 2.3: Do not design content in a way that is known to cause seizures^c^ [23]. Guideline 2.4: Provide ways to help users navigate, find content, and determine where they are^c^ [23]. Guideline 9: Provide access to content through a variety of navigation mechanisms, including sequential navigation, direct navigation, searches, and structured navigation^c^ [44]. Guideline 10: Provide information that will help the user understand browsing context^c^ [44]. Guideline 1: Ensure that the user can interact with the user agent (and the content it renders) through different input and output devices^c^ [44]. Guideline 5: Ensure that the user can control the behavior of viewports and user interface controls, including those that may be manipulated by the author (eg, through scripts)^b^ [44]. Guideline 2: Ensure that users have access to all content, notably conditional content that may have been provided to meet the requirements of the Web Content Accessibility Guidelines 1.0^b^ [44]. Guideline 3: Ensure that the user may turn off rendering of content (eg, audio, video, scripts) that may reduce accessibility by obscuring other content or disorienting the user^c^ [44]. Guideline 4: Ensure that the user can select preferred styles (eg, colors, the size of rendered text, and synthesized speech characteristics) from choices offered by the user agent. Allow the user to override author-specified and user agent default styles^c^ [44]. Guideline 7: Ensure that the authoring tool is accessible to authors with disabilities^c^ [43]. Guideline 1: Support accessible authoring practices^c^ [43]. Guideline 3: Support the creation of accessible content^c^ [43].

^a^Facilitation measure derived from expert opinion of researcher(s) conducting a study.

^b^Facilitation measure derived from empirical evidence.

^c^Facilitation measure derived from working group of experts.

**Table 5:** Barriers and facilitation measures categorized by the ‘robust’ foundational principle of Web accessibility.

Barrier	Facilitation measure
	Guideline 3: Support the creation of accessible content^a^ [43]. Guideline 2: Generate standard markup^a^ [43]. Guideline 4: Provide ways of checking and correcting inaccessible content^a^ [43]. Guideline 6: Implement interoperable interfaces to communicate with other software (eg, assistive technologies, the operating environment, and plug-ins)^a^ [44]. Guideline 8: Support the accessibility features of all implemented specifications. Implement W3C Recommendations when available and appropriate for a task^a^ [44]. Guideline 7: Observe operating environment conventions for the user agent user interface, documentation, input configurations, and installation^a^ [44]. Guideline 1: Ensure that the user can interact with the user agent (and the content it renders) through different input and output devices^a^ [44]. Guideline 4.1: Maximize compatibility with current and future user agents, including assistive technologies^a^ [23].

^a^Facilitation measure derived from working group of experts.

### Synthesis of Results

#### Categorization of Results by Foundational Principles of Web Accessibility

The identified barriers and facilitation measures were categorized according to the foundational principles of Web accessibility that was proposed by the W3C and are summarized in Tables 2 and 5 —additional tables organized by categories can be requested. Each identified barrier and facilitation measure was sorted into multiple categories if applicable. The barriers resulted in 3 categories as none were assigned to the robust category: operable (n=26); understandable (n=16); perceivable (n=4). The facilitation measures resulted into 4 categories: operable (n=35); understandable (n=49); perceivable (n=26); and robust (n=8).

Some studies paired a barrier with a corresponding facilitation measure, and other studies did not. The former was categorized based on the barrier, and the latter was categorized based on the specific barrier or facilitation measure that was not paired. Linking barriers that were not paired with a corresponding facilitation measure was beyond the scope of this review. A synthesis of Tables 2 and 5 is presented in the following section.

##### Operable

Identified barriers and facilitation measures (n=61) in this category gave most coverage to depression (49%), followed by bipolar disorder (43%), anxiety (41%), schizophrenia (39%), mental disorders (34%), schizoaffective disorder (20%), SMI (16%), and substance use disorder and other psychotic disorders equally (7%).

Barriers reported by included studies are primarily related to poorly designed navigational elements (eg, content filters), difficulties with fine motor coordination (eg, clicking small radio buttons, operating computer mouse, scrolling), poorly designed pages with time-limited response forms, too much information, and unoptimized components that contribute to slow webpage loading times.

Facilitation measures derived from empirical evidence gave guidance on design involving a reduction in the number of clicks needed to select options, an increase in buttons sizes, and websites that feature a shallow hierarchical structure and allows for personalization. Facilitation measures based on the expert opinion of researchers conducting studies suggest that websites should incorporate efficient content filters with intuitive navigation and permit users to browse at their pace.

Most facilitation measures recommended by the 3 included guidelines were focused on increasing users’ control. This involved providing users with enough time, alternative methods and information presentation styles, and instruction to interact with content. Other measures recommended that authoring tools must be accessible, promote accessible practices, and support the creation of accessible content.

##### Understandable

Most of the 64 identified barriers and facilitation measures in this category addressed depression (61%), schizophrenia (45%), anxiety (41%), mental disorders (34%), schizoaffective disorder (31%), and bipolar disorder (27%). However, SMI (3%) received considerably less coverage, and no barriers and facilitation measures were recorded for substance use disorder and other psychotic disorders in this category.

Included studies revealed barriers that included the use of complicated and excessive content, distracting and confusing design, and complex and overindulgent website functions (eg, excessive advertising and complicated purchasing processes). Facilitation measures derived from empirical evidence heavily focus on increasing the clarity of website content by ensuring only necessary information is shared and provided at a low reading level with no abbreviations and unfamiliar phrasing. Facilitation measures based on expert opinion focus more on the presentation and organization of website content. For example, they recommend the usage of alternative information formats, explicit labels that use concrete sentences to describe content and instructions, organizing content by importance, and forming meaningful content groups.

Facilitation measures from the 3 included guidelines recommend ways to help make content readable and understandable by ensuring abbreviations are expanded, reading level is appropriate, and providing explanations for any jargon used among other things. It was also recommended that several features should be incorporated into Web authoring tools: accessibility solutions in the design, mechanisms to correct inaccessible content and those that support accessible authoring practices.

##### Perceivable

Most of the 30 identified barriers and facilitation measures in this category targeted people with mental disorders (40%), depression (33%), anxiety and schizophrenia equally (30%), schizoaffective disorder (27%), bipolar disorder (17%) substance use disorder, and other psychotic disorders (3%). No barriers and facilitation measures were recorded for SMI in this category.

Identified barriers point to difficulties with reading small font, recognizing icons, and locating information. Facilitation measures derived both from empirical evidence and the expert opinion of researchers conducting studies recommend that links and other navigational elements should be easily recognizable, and use of images must be purposeful.

Facilitation measures recommended by the 3 included guidelines were predominantly focused on providing alternative content options and personal configurations for content. Other measures, all originating from the Authoring Tool Accessibility Guidelines1.0, generally recommend that authoring tools and practices must be accessible and support the creation of accessible content.

##### Robust

This category only contains facilitation measures from 1 of the 3 included guidelines, and no barriers were identified. All identified facilitation measures target PwMD. Recommended facilitation measures largely promote compatibility between user agents, authoring tools and Web content, and assistive technologies. The suggested methods to do this involve providing ways of checking and correcting inaccessible content within authoring tools and mainly adhering to standard markup, relevant W3C recommendations, and operating environment conventions.

#### Summary of Facilitation Measures Recommended by Studies

Facilitation measures recommended by studies were summarized into a group of 20 from 59 recommendations and are summarized in Table 6. Table 6 does not list or arrange summarized facilitation measures in any particular order. Nine of the summarized facilitation measures were the result of empirical work and 11 from the expert opinion of researchers.

**Table 6:** Summary of facilitation measures recommended by studies.

Derived from empirical evidence	Derived from expert opinion of researcher(s)
Provide instructions on how to change between different page views.	Provide intuitive navigation and ensure information filters and search functions work.
Build websites with a minimal number of layers.	Provide explicit instructions on how to use the website.
Provide legible font and perceivable buttons and links.	Use simple and familiar language with no abbreviations.
Comprehensively list hyperlinks surrounding a given topic.	Allow users to progress through the system at their own pace.
Allow for personalization or getting the best fit for the user.	Use graphics and colors sparingly and meaningfully.
Use of different media and technological additions (eg, reward logo or bookmark functionality).	Provide several options (eg, mouse, keyboard arrows, touch screen) to assist users with navigation.
Use attention-grabbing and not boring design.	Provide resources in video and audio format.
Use simple and familiar language.	Use legible font and sufficiently large buttons
Use an ample number of images and visual aids.	Use a simple design with webpages that are pleasing to the eye and easy to read.
	Meaningfully group information.
	Use a minimal amount of content.

## Discussion

### Principal Findings and Comparison With Prior Work

The 13 studies that could be included in this review support preexisting views [20,21] that there is little research on the barriers PwMD experience when using digital technology and facilitation measures used to address such barriers. Despite being few, included studies and guidelines give valuable insight into what is known and where knowledge gaps lie.

### Barriers People With Mental Disorders Encounter When Using Digital Technologies

People with mental disorders encounter a wide range of barriers when using the Web that makes it difficult for them to perceive, understand, and operate this tool along with content contained therein. Most barriers result from distracting and confusing design, complicated content and website functions, an overabundance of information, and a high-demand for good fine-motor skills and rapid information processing. Persons affected by other conditions associated with cognitive dysfunction have also been known to experience many of these barriers as indicated by Web design guidelines [47].

However, included barriers were related to neurocognitive dysfunction—impaired attention, processing and responding to information slowly and problem-solving—and none were associated with sociocognitive deficits—impaired affect regulation and difficulty processing emotional cues. This is possibly due to affective measurements being overlooked by researchers of included studies.

Barriers were predominantly identified using qualitative research methods and to a lesser extent mixed and quantitative methods. Identified barriers were often not well stated—not including details about the particular user category affected, disability type, hindered activity or task, and how it is hindered—and there was no indication of how restrictive barriers were or how often particular groups of participants encountered them. This can contribute to the development of tentative and inconclusive recommendations that may not be helpful.

### Recommended Facilitation Measures

Studies recommended facilitation measures that contribute towards ensuring the use of intuitive navigation, correctly functioning features, simple language, explicit, consistent and easy-to-detect website components, organized content, a flat hierarchical content structure, multimedia formats, and easy-to-operate functions. Facilitation measures recommended by included guidelines focused on improvement strategies that ensure websites are sufficiently operable, understandable, perceivable, and robust. Given the overlap in barriers, it was correctly anticipated that identified facilitation measures would also be mostly in agreement with recommendations for other conditions associated with cognitive deficits.

Facilitation measures were largely developed based on the opinion of researchers conducting studies and consensus among members of international working groups of experts in the area of accessibility. Some researchers [38] disagree with this approach because it does not involve empirical research with people affected by the particular condition when finding ways to meet their needs. However, facilitation measures derived from empirical work were similar to those based on the opinion of researchers conducting studies. Nonetheless, as shown in *Results* section, more focus was placed in different areas for 2 of the 3 principles under which facilitation measures were categorized. Facilitation measures recommended by included guidelines addressed problem areas, whereas other facilitation measures targeted specific barriers.

Facilitation measures were seldom linked to barriers. For example, no facilitation measures recommended by included guidelines had barriers associated with them. Consequently, many facilitation measures were recommended without validation and in a way that makes future validation difficult. This poses a challenge when selecting facilitation measures to address a particular barrier and attempting to increase the effectiveness of a particular facilitation measure.

### Coverage of Mental Disorders

As schizophrenia is associated with more severe cognitive deficits than other conditions [48,49] and many participants were also recruited from institutional settings, it was foreseeable that most studies in the area would involve people affected by these 2 conditions. Good cognitive ability is very important when using the Web [38], and the deficits associated with these conditions can put this population at high risk of encountering barriers when using digital technologies such as the Web. Although people affected by depression, anxiety, and bipolar disorder are believed to experience less severe cognitive deficits than those affected by schizophrenia [15], these conditions received similar coverage by included studies. This is possibly due to these conditions being common and the debilitating impact they could still have on the lives of people affected.

### Coverage of Digital Technologies

The overwhelming focus on websites out of many digital technologies demonstrates the heavy importance placed on the Web for its usefulness for PwMD. It also acknowledges that there is a need to further optimize Web-based resources. A single 1998 study [35] did not focus on websites but on a multimedia application. This is not surprising as the Web was not widely adopted during that time, but such applications were common.

### Types and Suitability of Study Designs

Qualitative methods were suitably adopted for most included studies because they sought to describe and explore technology usage and design for PwMD. The 3 other studies [37-39] investigated the effectiveness of design elements for PwMD and appropriately used quantitative usability testing methods.

It is acknowledged that more granular analysis and reporting of results by mental disorders in studies that involved people with more than 1 MD could potentially reveal a slightly different result. All studies except 3 [37,39-41] noted the classification of MD used when recruiting participants, and this makes it challenging to perform comparisons between results of similar studies and mental disorders and to confidently link results to classifications.

Included studies raise concerns about a bias toward Western culture owing to an absence of research conducted with participants from other cultures. Multicountry studies (eg, [50,51]) have established that culture helps shape technology usage to a great extent.

Participants in included studies ranged widely in age from 18 to over 75 years, and the experiences between younger and older participants were rarely compared or separated. It is important to account for age because it plays a significant role in determining the types of barriers individuals experience when using technology [52,53].

### Recency of Research

Findings show that more accessibility and usability research involving PwMD have been done in the last 5 years (10) compared with previous times (3). Considerably more research was done during the same period as revealed by a keyword search of several databases (ie, MEDLINE, PsycARTICLES, CINAHL, Library, Information Science and Technology Abstracts, Computers and Applied Sciences Complete, and ACM Digital Library) for Web or information and communications technology or digital accessibility or usability and visual (139), mobility (64), cognitive and learning (34), and auditory (17) impairments. This suggests that activity in the area is increasing but not at a rate comparable to similar research done with other populations. Included accessibility guidelines were dated. However, version 2.0 updates for Authoring Tool Accessibility Guidelines and User Agent Accessibility Guidelines are almost stable and referenceable versions that will likely be W3C Recommendations and new Web standards [54,55].

### Limitations

Although the literature search was conducted in many databases, results were limited to publications in English. However, no publications were later excluded based on this restriction. Included publications were not limited to those involving empirical work because preliminary searches indicated a paucity of research focusing on the area. As a result, international guidelines were included in the review. However, these guidelines are based on consensus among many experts and not empirical work, which allows for more valid conclusions. Moreover, although identified barriers found in studies were the result of empirical work, not all facilitation measures identified by studies were empirically validated. Nonetheless, as mentioned in *Discussion* section, empirically derived facilitation measures were similar to those based on the expert opinion of researchers conducting studies and were not in conflict with facilitation measures recommended by international guidelines.

Most of the included studies did not use a structured diagnostic classification (eg, ICD or DSM), and this has repercussions for our conclusions being tied to a diagnosis. For instance, it cannot be said unequivocally that persons with a particular diagnosis (eg, depression) experience a certain barrier as reported by those studies that did not use a structured diagnostic classification. Care was also taken to avoid making strong conclusions based on the small number of included studies (13), and it is advised that findings should be interpreted with this in mind.

### Implications and Recommendations for Practice and Future Research

Web professionals can now consult a full compilation of research and guidelines–based barriers and facilitation measures relevant to PwMD when developing and optimizing Web-based resources. This will raise awareness of PwMD’s needs when using the Web among Web professionals and potentially stimulate further discussion and action within the profession.

The body of research is in need of significant development, and it is too early to make meaningful conclusions on any particular MD, especially based on high-risk symptomatology. For future research, priority should be given to investigating all mental disorders initially. More research in the area is therefore required especially for mood, anxiety, dissociative, somatic, eating, sleep, impulse control, and personality disorders as these have attracted little or no attention.

In agreement with [56-58], an increased effort is needed to investigate the accessibility of technological innovations and health systems. This should be done in a more systematic way with clinically diagnosed samples to obtain conclusive evidence about what barriers exist and how they can be removed. This would involve ensuring each barrier is well stated along with an indication of the level of restriction it causes and frequency of occurrence among the particular user group. Validating strategies targeting the removal of barriers before recommending them as facilitation measures would also be helpful.

Additional actions could be taken by researchers to further develop this area of work. Incorporating valid measures for sociocognitive impairment allows for a more comprehensive evaluation of accessibility for PwMD. It would be important to know if there are cultural differences in the barriers encountered, the level of restriction a particular barrier causes, and/or the frequency of its occurrence. Accessibility studies could also consider a wider range of websites—social networking, e-commerce, education, health—and not just websites targeting PwMD to ensure all aspects of Web usage are investigated.

### Conclusions

Indeed, PwMD encounter barriers on the Web, and attempts have been made to remove or reduce these barriers. To the best of our knowledge, these results represent the first attempt to consolidate information on all barriers and facilitation measures investigated for PwMD when using digital technologies in a systematic way. However, it must be taken into consideration that only 13 studies and 3 guidelines meeting the inclusion criteria were identified. These findings also highlight the dire need for more rigorous research to be exhaustive and to have a larger impact on improving the Web for PwMD.

## Appendix

Multimedia Appendix 1 Summary of search concepts and terms.

## Abbreviations

MD: mental disorders
PwMD: people with mental disorders
SMI: severe mental illness
USA: United States of America
W3C: World Wide Web Consortium
