# Supplementary material for: Barriers and Facilitation Measures Related to People With Mental Disorders When Using the Web: A Systematic Review
Source: J Med Internet Res. 2016 Jun 9;18(6):e157. doi: 10.2196/jmir.5442 (PMC4919553; doi:10.2196/jmir.5442)
Supplement: Multimedia Appendix 1 [file jmir_v18i6e157_app1.pdf]

Multimedia Appendix 1. Search concepts and terms.

Concepts	Search Terms
Web Accessibility	accessibility, design for all, universal design, inclusive design, barrier free, accessible design, web accessibility, usability
Mental Disorders	mental disorder*, mental illness*, mental health, mentally ill person*, mental* ill*, schizophrenia, schizoaffective, psychosis, psychotic, depression, depressive, mania, manic, neurosis, anxiety, memory impairment, cognitive deficit, cognitive decline, cognitive impairment*, cognitive problem, cognitive disability, psychoneurosis, post-traumatic stress, posttraumatic stress, emotional trauma, bipolar, mood disorder, affective disorder, neurotic disorder, stress-related disorder, delusion, personality disorder, emotional disorder, emotional disability
Digital Technologies	computer systems, digital technology, computer, cyberspace, electronic, electronic mail, email, e-mail, internet, internet-based, net, online, web, web-based, world wide web, www, phone, telephone, smart phone, cell phone, mobile phone, cellular phone, mobile, short message service, sms, texting, smart device, text messaging, technology
